# Supplementary material for: Content-rich biological network constructed by mining PubMed abstracts
Source: BMC Bioinformatics. 2004 Oct 8;5:147. doi: 10.1186/1471-2105-5-147 (PMC528731; doi:10.1186/1471-2105-5-147)
Supplement: Additional File 5 — The original Chilibot query results of the term "long-term potentiation (LTP)" and 22 other terms, limiting the latest references analyzed to the years 1990, 1995, 2000, and 2004. [file 1471-2105-5-147-S5.bz2 › chilibotAdditionalFile5/ltp2000/html/CAMKIV.html]

 


**CAMKIV** (Input: CAMKIV ) 

---


**Google Searches:** Entire Web  | EDU domain only  | PDF files only

.

**External Links:** OMIM |
LocusLink
| Swissprot
| GeneCards

  
**Maps of CAMKIV**

Simple
Complete
graph in
radiant
tree
square
layout.

**New Hypothesis !**

**Synonyms** 

- camkiv   [PubMed]

**Synopsis**

- Our results suggest that **CaMKIV** may represent a physiologically relevant CREB kinase in T cells.  Mol Endocrinol, 1997 
  [23]

- Collectively, the data suggest that **CaMKIV** may be involved both in preventing apoptosis during T cell development and in the early cascade of events that is required to activate the mature T cells in response to a mitogenic stimulus.  Recent Prog Horm Res, 1997 
  [20]

- These results support an important role for **CaMKIV** Gr in calcium regulated neuronal gene transcription and synaptic plasticity and suggest that the contribution of other signaling pathways may spare spatial memory of **CaMKIV** Gr deficient mice.  J Neurosci, 2000 
  [20]

- In studying the signaling pathways by which synaptic inputs control the phosphorylation state of cyclic AMP responsive element binding protein CREB and determine expression of CRE regulated genes, we found two important calcium calmodulin CaM regulated mechanisms in hippocampal neurons a CaM kinase cascade involving nuclear **CaMKIV** and a calcineurin dependent regulation of nuclear protein phosphatase 1 activity.  Cell, 1996 
  [16]

- Notably, CD28 ligation activated p38 MAPK and **CaMKIV**, the kinases stimulated by CD3 engagement, suggesting that CD28 acts by increasing the activation extent of p38 MAPK and **CaMKIV**.  J Immunol, 2001 
  [16]

- We further showed that **CaMKIV** could be involved in the calcium dependent expression of the immediate early gene c fos, probably via phosphorylation of the transcription factor CREB.  Biochim Biophys Acta, 1996 
  [16]

- These results indicate that the activation of **CaMKIV** and CREB are necessary to establish a late phase of cerebellar LTD.  Neuron, 1999 
  [14]

- These results outline a virus regulated mechanism involving **CaMKIV** Gr which promotes transition from latency to productive viral replication in response to calcium mobilizing extracellular signals.  J Virol, 1997 
  [14]

- The focus of this review is to describe the biochemical regulation of **CaMKIV** and examine how **CaMKIV** activates transcription in response to calcium in both cell and animal models.  Biometals, 1998 
  [10]

- Therefore, several signaling molecules such as protein tyrosine phosphatases, PP2A **CaMKIV** and Ras are required for hPTP J transcription in Jurkat and Molt 4 cells.  Biochim Biophys Acta, 1999 
  [10]

- These findings reveal an intracellular signaling mechanism whereby a protein serine threonine kinase **CaMKIV** is regulated by a tightly associated protein serine threonine phosphatase PP2A .  Science, 1998 
  [10]

- When Ser142 was mutated to alanine, CREB was activated by CaMKII, as well as by **CaMKIV**.  Genes Dev, 1994 
  [9]

- In the cytoplasm, there is extensive cross talk between CaMKK, **CaMKIV** and other signaling cascades, including those that involve the cAMP dependent kinase PKA, MAP kinases and protein kinase B PKB.  Trends Biochem Sci, 1999 
  [7]

- Exposure of neurons to BDNF stimulates CREB phosphorylation and activation via at least two signaling pathways by a calcium calmodulin dependent kinase IV **CaMKIV** regulated pathway that is activated by the release of intracellular calcium and by a Ras dependent pathway.  Neuron, 1997 
  [7]

- The **CaMKIV** mediated gene induction requires the activity of CREB ATF family members but is independent of PKA activity.  Mol Cell Biol, 1994 
  [7]
